# EFFECTS OF PHYSICAL ACTIVITY AND CVD RISK ON MEMORY CONCERNS: RESULTS FROM THE NATIONAL HEALTH INTERVIEW SURVEY

**DOI:** 10.1093/geroni/igae098.3479

**Published:** 2024-12-31

**Authors:** Joel Anderson, Robin Harris, Jennifer Smith

**Affiliations:** University of Tennessee-Knoxville, Knoxville, Tennessee, United States; University of Tennessee, Knoxville, Tennessee, United States; University of Tennessee, Knoxville, Knoxville, Tennessee, United States

## Abstract

Common pathways exist between cardiovascular and cerebrovascular diseases in aging adults; therefore, this study examined the associations between the frequency of physical activity (PA) and memory issues in older adults with or at risk for cardiovascular disease (CVD). Data from the 2022 National Health Interview Survey were used to examine the influence of CVD risk and physical activity on self-reported experiences of memory challenges using logistic regression. The sample (N = 13,432) was limited to adults ≥50 years of age. Most of the sample was female, White, married/partnered, and reported a good to excellent health status. Roughly a quarter (24.9%) reported some difficulty remembering or concentrating while the majority (89.1%) had some level of increased CVD risk based on Framingham criteria. Half of the sample (51%) did not meet the current PA guidelines for older adults. After controlling for age, sex, race, ethnicity, and region, those with a poor or fair health status (OR = 3.24, CI = 2.94–3.56, p < 0.001), increased CVD risk (OR = 1.26, CI = 1.09–1.47, p = 0.002), or who did not meet current PA guidelines (OR = 1.67, CI = 1.46–1.90, p < 0.001) more frequently reported challenges with remembering or concentrating. Given these findings, further research is warranted into the role of PA in memory issues, particularly for those at risk for CVD. Following guidelines for PA for these older adults may be helpful.

